# Anxiety, depression and psychological well-being in a cohort of South African adults with Type 2 diabetes mellitus

**DOI:** 10.4102/sajpsychiatry.v22i1.935

**Published:** 2016-07-08

**Authors:** Samantha Ramkisson, Basil J. Pillay, Benn Sartorius

**Affiliations:** 1 Department of Behavioural Medicine, School of Nursing and Public Health, University of KwaZulu-Natal, South Africa; 2 Department of Public Health Medicine, School of Nursing and Public Health, University of KwaZulu-Natal, South Africa

## Abstract

**Background:**

The prevalence of diabetes mellitus (DM) has increased at alarming rates globally. South Africa has the second highest number of people in Africa living with DM, with prevalence rates being among the top five countries in Africa. Accordingly, psychological issues associated with DM have been a growing focus of attention. Studies have found that patients with DM have elevated levels of anxiety and depression, and decreased levels of well-being. In South Africa, there is a paucity of studies on the psychological issues associated with DM.

**Objectives:**

The aim of this paper was to explore the prevalence and association of anxiety, depressive features and psychological well-being in patients with Type 2 DM.

**Method:**

In a cross-sectional survey, patients with Type 2 DM were recruited from public and private facilities. The Hospital Anxiety and Depression Scale (HADS), the General Health Questionnaire (GHQ-28) and WHO-5 Well-being Index (WHO-5) were administered.

**Results:**

Four hundred and one participants completed the questionnaires. On the WHO-5, 277 (69%) reported good well-being, while 124 (31%) indicated poor well-being and were considered at risk for depressive features. On the HADS, 186 (46%) had mild-to-severe depressive features and 128 (32%) had mild-to-severe anxiety. There was a strong negative correlation between the WHO-5, HADS and General Health Questionnaire (GHQ) scales, which indicated that an increase in anxiety and depressive features decreased psychological well-being.

**Conclusion:**

Health-care providers should identify and treat anxiety and depression as a standard part of diabetes care. Patients should also be referred to the appropriate mental health professional as part of the management of diabetes.

## Introduction

There has been an alarming increase in diabetes mellitus (DM) prevalence rates globally. The International Diabetes Federation (IDF) revealed that in 2014 the global prevalence rate was estimated to be 8.2%, with about 382 million people living with the disease. This figure is projected to increase to 592 million by 2035.^1^ The IDF projects that the global figure of 21.5 million people estimated to be living with diabetes in 2014 will increase dramatically to 41.5 million in 2035. In Africa, the prevalence rate is estimated to be 5.1%.^2^ South Africa has a prevalence rate of 9.27% and has the second highest number of people in Africa living with the disease – an estimate of 2.6 million.^3^ In 2012, DM was the fifth leading cause of death in South Africa and the third leading cause of death in KwaZulu-Natal.^4^

In recent years, much attention has been paid to the psychological well-being of patients with diabetes.^5^ The cross-national Diabetes Attitudes, Wishes and Needs (DAWN) study, conducted by Peyrot et al.^6^ in Asia, Australia, Europe and North America in 2005, found that 41% of patients with DM reported poor psychological well-being. Furthermore, they found that 61% – 72% of health-care providers reported that their patients with DM had psychological problems, which included depression, anxiety, stress and burnout. Karlsen et al.^7^ found that poor psychological well-being was more prevalent among people with Type 2 diabetes compared with people with Type 1 diabetes.

The high levels of poor psychological well-being thus become an important issue to be addressed in the management of diabetes. However, Pouwer et al.^8^ state that while psychological well-being is understood to be an important goal in diabetes management, often little attention is paid to addressing these psychological aspects of the disease.

Most studies using the Hospital Anxiety and Depression Scale (HADS) found that DM is associated with increased levels of anxiety and depression.^9,10,11,12^ Longitudinal studies done by Engum^13^ and Fisher et al.^14^ also found that patients with DM have elevated levels of anxiety and anxiety symptoms.

Anderson et al.^15^ and Ali et al.^16^ report that the odds of depression in diabetic groups were twice those of the non-diabetic comparison groups, and this did not differ by sex, type of diabetes, subject source or assessment method. Coexisting depression in people with DM is associated with decreased adherence to treatment, poor metabolic control, higher complication rates and decreased quality of life.^17^ As a result, patients with DM often experienced feelings of poor well-being, stress and anxiety.^18^ Studies also show that women report higher levels of depression, anxiety and stress compared with men.^12,19^

From the above discussion, it can be seen that there is a growing body of evidence to support the view that, in addition to the medical/pharmacological management of people with diabetes, the management of psychological, psychiatric and social aspects is also essential. This is necessary given the high levels of anxiety and depression found in people with diabetes.

In South Africa, there is a paucity of studies on the psychological issues associated with DM. We hypothesise that the presence of anxiety and depressive features decreases the well-being of patients with Type 2 diabetes. To our knowledge, a study of this nature has not been done in South Africa.

## Method

Patients with Type 2 DM were recruited from two public facilities and five private general medical practices situated on the North Coast of KwaZulu-Natal, South Africa.

### Participants

Patient volunteers, 18 years and older, diagnosed with Type 2 DM at least 6 months previously and able to speak either English or isiZulu, were included in the study. Patients who were intellectually impaired and/or who had serious medical complications that decreased their quality of life (e.g. amputations or dialysis) were excluded from the study. Four hundred and twenty-four participants were recruited over a period of 6 months. Twenty-three participants were excluded due to incomplete data. A total of 401 participants completed the questionnaires; this comprised 201 patients from the public health facilities and 200 patients from the private health facilities.

### Ethical considerations

Ethics approval was obtained from the Biomedical Ethical Research Committee of the University of KwaZulu-Natal. Consent was also obtained from the Provincial Department of Health and the managers of the public facilities. Written permission was also obtained from the private doctors to conduct the research at their practices.

### Procedure

A cross-sectional study design was used whereby participants were recruited consecutively until the required sample size was achieved. Trained research assistants, fluent in both English and isiZulu, explained the study in detail to patients awaiting their regular treatment appointments. Those who met the inclusion criteria were invited to participate. A written informed consent form was signed by the participant in their language of choice. The questionnaires were administered at the study sites by a trained research assistant. The participant’s diabetes type was confirmed by the diabetes nurse educators and information from the medical file.

### Quantitative instruments

This article is part of a larger study on psychological well-being and Type 2 diabetes which aims to explore the perceptions of support and the biopsychosocial factors associated with well-being in adults with Type 2 diabetes. This article reports on data of the HADS, the General Health Questionnaire (GHQ) and the WHO-5 Well-being Index (WHO-5).

The HADS, developed by Zigmond and Snaith^20^ is a self-report questionnaire designed to detect anxiety and depressive states. It has 14 items with two subscales measuring symptoms of depression and symptoms of anxiety. Bjelland et al.^21^ found that the HADS performed well in assessing the symptom severity of anxiety disorders and depression in psychiatric primary-care patients and in the general population.^11,22^ Cronbach’s alpha values for this instrument range from α = 0.40 to α = 0.70.^21^ The Cronbach’s alpha for this study was 0.83 for the HADS Anxiety scale and 0.79 for the HADS Depression scale.

The GHQ-28, developed by Goldberg, is a 28-item measure of emotional distress.^23^ There are four subscales with the 28 items distributed evenly across each. The four subscales are somatic symptoms, anxiety and insomnia, social dysfunction, and severe depression. Internal reliability ranges from 0.78 to 0.95.^24^ In this study, the Cronbach’s alpha ranged from 0.86 to 0.92.

The WHO-5 has five items and covers positive mood (good spirit, relaxation), vitality (being active, waking up fresh and rested) and general interests (being interested in things). The degree to which these feelings were present in the previous 2 weeks is scored on a 6-point Likert scale ranging from 0 (not present) to 5 (constantly present). The scores are summarised, with raw scores ranging from 0 to 25 and then transformed to 0–100 by multiplying by 4 with higher scores signifying better well-being.^25^ A score of < 52 suggests poor emotional well-being and is a sign for further testing. A score ≤ 28 is indicative of depression. The Cronbach’s alpha of the WHO-5 in this study was 0.89.

All questionnaires were translated into isiZulu by an accredited isiZulu translator. The documents were back-translated into English by an isiZulu educator.

### Data analysis

The data were analysed using STATA version 13.0.^26^ The following tests were used: *t*-test and Wilcoxon rank-sum test for comparing means of continuous data across two groups (e.g. public vs private sectors), the Chi-square (χ^2^) test or Fisher’s exact for cross-tabulations of categorical variables, and the one-way ANOVA or Kruskal–Wallis equality-of-populations rank test to compare means (or summed ranks) of continuous variables across three or more groups.

In order to detect a small effect size (Cohen’s *d* = 0.28)^27^ when assessing differences in mean age, for example, by public/private sector or anxiety, depression and psychological well-being with 80% power (1-β [Type 2 error probability]) and 95% confidence (or 5% α error probability [Type 1]) using a student’s *t*-test, a sample size of 400 participants (200 in each group) would be required.

Bivariate and multivariable ordinal logistic regression was performed to identify factors associated with ordinal well-being category after adjustment for confounders and the influence of other risk factors. The Brant Test of Parallel Regression Assumption was run to ensure that the proportional odds assumption was not violated. Potential multiple colinearity between independent variables within the model was assessed using Variance Inflating Factors (VIF). An adjusted *p* < 0.05 was deemed statistically significant.

## Results

The demographic characteristics of the study sample are presented in Table 1. The mean age was M = 53.7 years (s.d. = 10.7), with a similar mean age in the public sector (M = 53.5 years; s.d. = 10.3) and private sector (M = 53.83; s.d. = 11.2; *p* = 0.761). The mean age of females (M = 52.83; s.d. = 10.54) was marginally statistically younger compared with males (M = 54.75; s.d. = 10.96; *p* = 0.054). The average duration of the disease for the entire sample was 10.3 years (s.d. = 7.9). This did not vary by sector, with a median duration of 8 years (IQR = 4–14) for the public sector and 8 years (IQR = 4–13) for the private sector.

**TABLE 1:** Demographic characteristics of the study sample by health sector.

Variable	Public Sector	Private Sector	Total	*p*-value*
		
	*n* (%) *n* = 201	*n* (%) *n* = 200	*n* (%) *n* = 401	
Mean Age	53.50 (10.3)	53.83 (11.2)	53.70 (10.7)	
**Gender**				
Male	55 (27.40)	103 (51.50)	158 (39.4)	< 0.001
Female	146 (72.60)	97 (48.50)	243 (60.6)	
**Marital Status**				
Never married	46 (22.90)	7 (3.50)	53 (13.2)	< 0.001
Married	116 (57.70)	160 (79.60)	276 (68.8)	
Separated/Divorced	14 (7.00)	10 (5.00)	24 (6)	
Widowed	25 (12.40)	23 (11.40)	48 (12)	
**Race**				
White	2 (1.00)	22 (10.90)	24 (6.00)	< 0.001
Black	85 (42.30)	15 (7.50)	100 (24.9)	
Coloured	3 (1.50)	0 (0.00)	3 (0.75)	
Indian	111 (55.22)	163 (81.50)	274 (68.33)	
**Educational**				
Some high school or Grade 12	187 (93.03)	117 (58.50)	304 (75.81)	< 0.001
Post Grade 12	14 (6.97)	83 (41.50)	97 (24.19)	
**Employment**				
Employed	56 (27.86)	127 (63.50)	183 (45.64)	< 0.001
Unemployed	94 (46.77)	20 (10.00)	114 (28.43)	
Retired or homemaker	51 (25.37)	53 (26.50)	104 (25.94)	

* Chi-squared (χ2) test or Fisher’s exact test (if expected cell count < 5) used to compare categorical variables by public/private sectors.

In Table 1, there were significant differences for selected socio-demographic characteristics when comparing the private and public sectors. Females attended the public sector facilities almost three times more than males (*p* < 0.001). More black African patients attended the public sector services, whereas private sector services were predominantly accessed by white patients (*p* < 0.001). The majority of participants in the private sector were married (80%); private sector participants also had more formal education (41.50%) and more were in full-time employment (47%). The highest prevalence of DM was among the Indian racial group (68.1%).

On the WHO-5, 277 (69%) of the sample endorsed good well-being, while 124 (31%) reported poor well-being and were thus considered at risk for depression (see Table 2). More females (43; 17.70%) endorsed poor well-being than males, while 44 (22%) of the public sector sample reported poor well-being. Furthermore, participants who were married (207) had better well-being levels (75%) compared with those who had never married (24; 45.28%). Also, Indian and white groups demonstrated better well-being levels compared to the black African and mixed racial groups. Participants who had a higher educational level (77; 79.38%) or were employed (136; 74.32%) also had better well-being levels.

**TABLE 2:** Levels of well-being by gender, sector, marital status, ethnicity, education and employment.

Variable	Well-being category			*p*-value*	Odds ratio	CI (95%)

	Normal *n* (%)	Low mood *n* (%)	Depressed *n* (%)			
Well-being of the total study sample	277 (69.08)	64 (15.96)	60 (14.96)			
**Gender**						
Male	124 (78.48)	17 (10.76)	17 (10.76)	0.004	1	
Female	153 (62.96)	47 (19.34)	43 (17.70)		0.48	(0.30-0.75)
**Sector**						
Public	118 (58.71)	39 (19.40)	44 (21.89)	< 0.001	0.36	(0.23-0.55)
Private	159 (79.50)	25 (12.50)	16 (8.00)		1	
**Marital Status**						
Never married	24 (45.28)	13 (24.53)	16 (30.19)	< 0.001	1	
Married	207 (75.00)	38 (13.77)	31 (11.23)		3.55	(2.01-6.30
Separated/Divorced	12 (50.00)	7 (29.17)	5 (20.83)		1.32	(0.54-3.23)
Widowed	34 (70.83)	6 (12.50)	8 (16.67)		2.75	(1.24-6.10)
**Ethnic Groups**						
White	20 (80.00)	4 (16.00)	1 (4.00)	0.002	1	
Black	54 (54.00)	23 (23.00)	23 (23.00)		0.30	(0.1-0.85)
Coloured	1 (33.33)	1 (33.33)	1 (33.33)		0.15	(0.16-1.44)
Indian	203 (74.10)	36 (13.19)	35 (12.82)		0.70	(0.2-1.92)
**Educational Level**						
Some high school or Grade 12	200 (65.79)	50 (16.45)	54 (17.76)	0.013	1	
Post Grade 12	77 (79.38)	14 (14.43)	6 (6.19)		2.11	(1.23-1.92)
**Employment**						
Unemployed	60 (52.63)	25 (21.93)	29 (25.44)	< 0.001	1	
Employed	136 (74.32)	28 (15.30)	19 (10.38)		2.65	(1.64-4.29)
Retired or homemaker	81 (77.88)	11 (10.58)	2 (11.54)		3.10	(1.74-5.54)

CI, confidence interval.

* Chi-squared (χ^2^) test or Fisher’s exact test (if expected cell count < 5) used to compare categorical variables.

Mean scores comparisons of the HADS and the GHQ by gender and health sectors are displayed in Table 3. Based on the GHQ-Somatic scale, 159 (40%) participants had elevated somatic symptoms; on the GHQ-Anxiety/Insomnia scale, 149 (37.16%) reported high levels of anxiety. On the GHQ-Social Dysfunction scale, 290 (72.32%) had high levels of social dysfunction, and on the GHQ-Depression scale 78 (19%) reported severe depressive symptoms. Females (M = 25.50; s.d. = 16.11) had significantly higher mean GHQ score compared to males (M = 18.40; s.d. = 12.80). The public sector mean GHQ score (M = 25.20; s.d. = 15.84) was significantly higher than the private sector (M = 20.16; s.d. = 14.30). The Social Dysfunction scale scores were elevated both for gender and health sector.

**TABLE 3:** Comparison of the HADS and GHQ mean scores for gender and health sector.

Variable	Gender					Sector					Total M (s.d.)	95% CI
	
	Male M (s.d.)	95% CI	Female M (s.d.)	95% CI	*p*-value*	Public M (s.d.)	95% CI	Private M (SD)	95% CI	*p*-value		
**HADS**												
Depression	6.46 (4.30)	(5.80-7.13)	8.08 (4.34)	(7.50-8.64)	< 0.001	7.90 (4.41)	(7.24-8.50)	7.02 (4.50)	(6.40-7.64)	0.060	7.44 (4.45)	(7.00-7.90)
Anxiety	5.22 (3.71)	(4.63-5.80)	6.10 (4.18)	(5.60-6.63)	0.031	6.50 (4.20)	(6.00-7.10)	5.03 (3.74)	(4.51-6.00)	0.060	5.75 (4.02)	(5.40-6.15)
**GHQ**												
Somatic	11.75 (3.91)	(11.13-2.40)	14.43 (4.95)	(13.80-5.10)	< 0.001	14. 10 (4.80)	(13.40-4.74)	12.70 (4.61)	(12.03-3.32)	0.003	13.37 (4.75)	(12.91-3.84)
Anxiety Insomnia	11.50 (4.51)	(10.77-2.20)	13.54 (5.20)	(12.88-4.20)	< 0.001	13.30 (5.13)	(12.60-4.01)	12.20 (4.87)	(11.50-2.83)	0.022	12.73 (5.03)	(12.24-3.22]
Social Dysfunction	14.15 (3.12)	(13.65-4.63)	15.17 (3.69)	(14.71-5.64)	0.022	15.44 (3.90)	(14.90-6.00)	14.09 (2.92)	(13.68-4.50)	< 0.001	14.77 (3.51)	(14.43-5.11)
Depression	9.01 (3.70)	(8.43-9.60)	10.30 (5.03)	(9.70-10.94)	0.006	10.35 (4.8)	(9.70-11.01)	9.24 (4.33)	(8.64-9.84)	0.016	9.80 (4.60)	(9.34-10.25)

**Total**	**18.40 (12.80)**	**(16.38-0.40)**	**25.50 (16.11)**	**(23.41-7.50)**	**< 0.001**	**25.20 (15.84)**	**(23.00-7.40)**	**20.16 (14.30)**	**(18.17-2.14)**	**0**.**001**	**22.70 (15.30)**	**(21.80-4.20)**

HADS, Hospital Anxiety and Depression Scale; GHO, General Health Questionnaire.

s.d., standard deviation; CI, confidence interval.

* Chi-squared (χ^2^) test or Fisher’s exact test (if expected cell count < 5) used to compare categorical variables.

Based on the HADS, 186 (46%) participants had mild-to-severe depressive features and 128 (32%) had mild-to-severe anxiety. Females (M = 8.08; s.d. = 4.34) had significantly higher scores on the HADS Depression scale compared to males (M = 6.46; s.d. = 4.30). The total mean score for depression scale was 7.44 (s.d. = 4.45), which was higher than the total mean scores for anxiety (M = 5.75; s.d. = 4.02). Although these scores are not diagnostic, they suggest the presence of symptoms of anxiety and depressive features.

The relationships between the WHO-5, the HADS and the GHQ scales were investigated using Spearman’s rank correlation coefficient (*r*) as shown in Table 4. There was a strong negative correlation between the WHO-5, HADS Depression, HADS Anxiety, GHQ-Somatic GHQ-Anxiety, GHQ-Social and GHQ-Depression scales. A moderate positive correlation was found between the HADS Depression, HADS Anxiety, GHQ-Somatic, GHQ-Anxiety and GHQ-Depression scales. There was a strong positive correlation between the GHQ-Anxiety and GHQ-Somatic scales (*ρ* = + 0.74, *p* < 0.05).

**TABLE 4:** Correlation between WHO-5, HADS and GHQ scales.

Scales	1	2	3	4	5	6	7
1. WHO-5	-						
2. HADS (De-pression)	−0.579*	-					
3. HADS (Anxie-ty)	−0.632*	0.662*	--				
4. GHQ (Somatic)	−0.537	0.613*	0.512*	--			
5. GHQ (Anxiety)	−0.575*	0.668*	0.558*	0.744*	--		
6. GHQ (Social)	−0.529*	0.463*	0.499*	0.530*	0.538*	-	
7. GHQ (Depres-sion)	−0.486*	0.614*	0.553*	0.539*	0.615*	0.485*	-

HADS, Hospital Anxiety and Depression Scale; GHO, General Health Questionnaire.

* *p* < 0.05

The mean scores for depression, anxiety and GHQ-Depression by well-being category are displayed in Table 5. Mean scores significantly increased from the normal group to the depressed group (*p* < 0.001). Pairwise comparisons in the HADS Depression category showed that there were significant differences between the normal and depressed groups, and the normal and low mood groups. For the HADS Anxiety and the GHQ-Depression scales, pairwise comparisons showed significant differences (*p* < 0.001) between low mood and depressed, normal and depressed, and normal and low mood. Among the depressed group in the well-being category, a large proportion (65%) on the HADS had moderate-to-severe depressive features. On the HADS Anxiety scale, 53% were in the moderate-to-severe depressed category of well-being. There was a significant association between the well-being categories, the HADS and the GHQ-Depression categories, where an increase in depression and anxiety scores indicated lower well-being (Fisher’s exact *p* < 0.001).

**TABLE 5:** HADS and GHQ-Depression categories compared to WHO-5 well-being categories.

Variable	Well-being category			Total	*p*-value

	Normal *n* (%)	Low mood *n* (%)	Depressed *n* (%)		
**HADS Anxiety**					
Normal	227 (81.95)	36 (56.25)	10 (16.67)	273 (68.08)	< 0.001*
Mild	41 (14.80)	18 (28.13)	18 (30.00)	77 (19.20)	
Moderate	9 (3.25)	8 (12.50)	20 (33.33)	37 (9.23)	
Severe	0 (0.00)	2. (3.13)	12 (20.00)	14 (3.49)	
Mean (s.d.)	5.80 (3.50)	10.30 (3.50)	12.20 (4.40)	7.44 (4.45)	< 0.001†
**Total**	**277**	**64**	**60**	**401**	
**HADS Depression**					
Normal	187 (67.51)	18 (28.13)	10 (16.67)	215 (53.62)	< 0.001*
Mild	62 (22.38)	14 (21.88)	11 (18.33)	87 (21.70)	
Moderate	27 (9.75)	23 (35.94)	19 (31.67)	69 (17.21)	
Severe	1 (0.36)	9 (14.06)	20 (33.33)	30 (7.48)	
Total	277	64	60	401	
Mean (s.d.)	4.20 (3.10)	7.50 (3.00)	10.90 (3.80)	5.75 (4.02)	< 0.001†
**Total**	**277**	**64**	**60**	**401**	
**GHQ-Depression**					
≥ 7	261 (94.22)	43 (67.19)	28 (46.67)	332 (82.79)	< 0.001*
< 7	16 (5.78)	21 (32.81)	32 (53.33)	69 (17.21)	
Mean (s.d.)	8.20 (2.60)	12.20 (5.50)	14.60 (6.10)	2.80 (4.59)	< 0.001†
**Total**	**277**	**64**	**60**	**401**	

s.d., standard deviation.

* Fisher’s exact test; †, Kruskal-Wallis equality-of-populations rank test.

HADS, Hospital Anxiety and Depression Scale; GHO, General Health Questionnaire.

After adjusting for age, gender, race and type of facility (Table 6), the following factors were significantly associated with lower odds (Odds Ratio: OR) of being in a better well-being category: participants attending the public sector (OR = 0.43; 95% CI: 0.23; 0.78), mild (OR = 0.44; 95% CI: 0.23; 0.85) moderate (OR = 0.21; 95% CI: 0.10; 0.44) or severe depressive features (OR = 0.07; 95% CI: 0.03; 0.22) compared with the normal category, as well as mild (OR = 0.42; 95% CI: 0.23; 0.77), moderate (OR = 0.17; 95% CI: 0.07; 0.40) or severe anxiety (OR = 0.11; 95% CI: 0.02; 0.61) compared with the normal category. Similarly, those with a GHQ-Somatic (OR = 0.74; 95% CI: 0.36; 1.51), GHQ-Anxiety (OR = 0.50; 95% CI: 0.23; 1.04), GHQ-Social Dysfunction (OR = 0.50; 95% CI: 0.22; 1.00) or GHQ-Depression (OR = 0.43; 95% CI: 0.22; 0.85) with a threshold score ≥ 7 were also significantly less likely to be in a better well-being category, following multivariable adjustment.

**TABLE 6:** Crude and adjusted analysis of factors affecting well-being.

Variable	*n*	Crude (univa-riate)			Adjusted (multivaria-ble)		
	
		OR	95% CI	*p*-value	OR	95% CI	*p*-value
**Characteristic**							
Age	401	1.03	(1.01-1.05)	0.006	1.02	(1.00-1.05)	0.087
Female gender	401	0.48	(0.30-0.75)	0.002	1.12	(0.44-1.36)	0.373
**Ethnicity**							
Black people	100	1 (reference)	-	-	-	-	-
White people	24	3.35	(1.18-9.51)	0.023	1.30	(0.34-4.94)	0.704
Indian	273	2.34	(1.48-3.72)	< 0.001	2.30	(1.23-4.27)	0.009
Mixed race	4	0.87	(0.07-3.99)	0.523	1.59	(0.40-6.30)	0.888
**Type of facility**							
Private	200	1 (reference)	-	-	-	-	-
Public	201	0.36	(0.23-0.55)	< 0.001	0.43	(0.23-0.78)	0.005
**Depression (The Hospital Anxiety and Depression Scale)**							
Normal	215	1 (reference)	-	-	-	-	-
Mild	87	0.36	(0.20-0.66)	0.001	0.44	(0.23-0.85)	0.015
Moderate	69	0.1	(0.06-0.18)	< 0.001	0.21	(0.10-0.44)	< 0.001
Severe	30	0.02	(0.01-0.04)	< 0.001	0.07	(0.03-0.22)	< 0.001
**Anxiety (The Hospital Anxiety and Depression Scale)**							
Normal	273	1 (reference)	-	-	-	-	-
Mild	77	0.21	(0.12-0.36)	< 0.001	0.42	(0.23-0.77)	0.006
Moderate	37	0.05	(0.02-0.11)	< 0.001	0.17	(0.07-0.40)	< 0.001
Severe	14	0.01	(0.00-0.04)	< 0.001	0.11	(0.02-0.61)	0.012
**General Health Ques-tionnaire Scales**							
Somatic	401	0.13	(0.85-0.21)	0.000	0.74	(0.36-1.51)	< 0.001
Anxiety	401	0.10	(0.63-0.17)	0.000	0.50	(0.23-1.04)	< 0.001
Social	401	0.20	(0.10-0.40)	0.000	0.50	(0.22-1.00)	< 0.001
Depression	401	0.09	(0.05-0.16)	< 0.001	0.43	(0.22-0.85)	0.015

## Discussion

The aim of this study was to explore the prevalence and the relationships between anxiety, depressive features and psychological well-being in adults with Type 2 DM in the public and private health sectors.

There were no differences between the public and private sectors in terms of the mean age and the average duration of the disease. There were significantly more females attending the health-care facilities compared to males.^28,29,30,31,32^ have also documented this trend. Women tend to be more active than men when seeking health-care and health-care information^33^ and tend to be more supportive of others’ health-care needs but lack the support they need when managing their own health.^32^

Participants of Indian race had the highest levels of DM, which supports research suggesting a higher prevalence of diabetes in this racial group.^34,35^ According to Seedat^34^ this is the result of insulin resistance, an unhealthy diet and physical inactivity. Black African participants (24.9%) had the next highest levels of DM. In recent years, the prevalence of DM has increased in black South Africans,^36,37^ due to a family history of DM, high systolic blood pressure, high total cholesterol^37^ and urbanisation, which is accompanied by a change of diet leading to raised levels of obesity.^36^

Among public sector participants, the unemployment rate was 46.77%, which is higher than the current national unemployment rate in South Africa (26.4%) and double the KwaZulu-Natal rate (23.6%).^38^ This suggests that patients who are unemployed understandably use the public health-care facilities, having no alternative avenue for care due to lack of finances. However, due to an overburdened health-care system, it is very likely that patients with DM do not receive adequate treatment and have to settle for suboptimal care.^39,40^ This, in turn, increases long-term complications and medical costs.

### Psychological well-being

Our findings support the view that a significant number of people with DM have poor psychological well-being. Poor psychological well-being negatively impacts a person’s ability to manage diabetes, decreases their adherence to medication and increases the risk of complications later in life.^41,42^ Self-management and adherence to medication are of paramount importance in achieving the prevention of diabetic complications, which is one of the core medical goals in the management of diabetes. The impact of poor psychological well-being in patients with diabetes has serious implications both medically and psychologically. This further highlights the need for the standard provision of psychological services to patients with DM.

This study showed that more women reported poor well-being compared with men. This is in keeping with the literature,^9,19,43,44,45^ suggesting that gender-role differentiation and the responsibilities of taking care of the family^19^ contribute to this. Furthermore, females may not have much social support in the management of the disease, although they are more likely to be supportive to others.

Another finding on well-being in this study was that married participants reported better well-being. According to Cohen et al.^46^ and Vlassoff,^32^ males with diabetes receive more support from their wives compared with the females with diabetes, who receive less support from their spouses. Supporting this view, the results show that participants who were never married had poorer psychological well-being.

There were differences on the report of well-being among the racial groups. It was found that white people and Indian groups reported better psychological well-being compared with the black racial group. Factors such as socio-economic status and educational level are likely to contribute to such differences. In South Africa, the black racial group has the highest rate of unemployment compared with the white and Indian groups,^47^ which limits access to adequate medical care. Greater awareness and knowledge about the disease improve compliance with medication regimes.^18,48^ Knowledge and information about DM can be obtained through the Internet; however, more than half of the population do not have Internet access.^49^ This further hampers knowledge and awareness about the disease.

### Anxiety and depressive features

The prevalence rates in our study were much higher compared with studies in developed countries like the United Kingdom and the United States^10,11,43,50^ and similar to studies in developing countries like Pakistan and China.^9,51^ Furthermore, in our study we found that the prevalence of depressive features was higher than that of anxiety. Anderson et al.^15^ and Ali et al.^52^ reported that people with Type 2 diabetes had elevated rates of depression. The high rates of anxiety and depressive features are a cause for concern and need to be taken into consideration by health-care providers when treating DM.

In keeping with literature, we found that females reported higher levels of depressive features compared with males.^9,12,15,43,53^ A contributory factor is the added burden of having a demanding chronic condition while having to performing gender role-specific tasks such as child-bearing and taking care of the family. As pointed out by Ngcobo and Pillay,^53^ the lack of resources for women in South Africa hinders or prevents early treatment and access. Improving women’s accessibility to health-care, and thereby their health, will appreciably impact on the general health status of families and communities.

### Psychological well-being, anxiety and depressive features

The strong negative correlation between the WHO-5, HADS Depression, HADS Anxiety, GHQ-Somatic, GHQ-Anxiety, GHQ-Social and GHQ-Depression scales indicates that an increase in anxiety and depressive features results in a decrease in psychological well-being. As expected, this finding supports our hypothesis that the presence of anxiety and depressive features decreases well-being. The strong positive correlation between the GHQ-Anxiety and GHQ-Somatic scales is understandable because patients with diabetes have many somatic complaints; these could lead to diabetic complications, thereby increasing anxiety and depressive levels. Pillay and Cassimjee^54^ support the view that there is a high incidence of depressive and anxiety disorders in patients who are seen in general practice; however, a large proportion of these are not identified. Studies by Wu et al.^55^ found that anxiety correlated positively with diabetic complications.

Our study found that patients attending the public sector, and patients who have symptoms of anxiety and depressive features, have lower levels of well-being. This highlights the need for health services to place greater emphasis on the psychological needs and treatment of patients with diabetes in the public health sector. Patients with diabetes should routinely undergo screening of their mental health status. Addressing psychological needs will improve medical outcomes, self-management and regimen adherence, thereby preventing long-term complications of diabetes.

### Limitations

Although the study aimed to be representative of all racial groups, some racial groups had small sample sizes. The cross-sectional design limits causal associations between anxiety, depressive features and psychological well-being. Self-administered questionnaires were used in this study, but a diagnostic interview remains the gold standard in research and clinical practice.

## Conclusion

The findings in this study supported our hypotheses about anxiety, depressive features and psychological well-being of patients with diabetes. These are important findings when considering the management of patients with diabetes. The findings suggest that for effective treatment of DM, health-care providers must be able to identify and treat anxiety and depression as common components of diabetes care. Routine screening for anxiety and depression is necessary for patients with diabetes. It is also suggested that patients be referred to the appropriate mental health professionals as part of the management of diabetes.
